# Biceps Tenotomy Does not Affect the Functional Outcomes of Patients Treated with Spacer Implantation Due to Massive Irreparable Rotator Cuff Tears

**DOI:** 10.2174/1874325001711011577

**Published:** 2017-12-29

**Authors:** Eran Maman, Ori Safran, Shaul Beyth, Gavriel Mozes, Assaf Dekel, Bernstein Michael, Ofir Chechik, Eliyahu Adar

**Affiliations:** 1 Shoulder Unit, Orthopedic Surgery Division Tel Aviv Medical Center, Sackler Faculty of Medicine, Tel Aviv University, Tel Aviv, Israel; 2 Orthopedic Surgery, Hadassah Medical Center, Jerusalem, Israel; 3 Advanced Orthopedic Center, Assuta Medical Center, Tel Aviv, Israel; 4 Yoseftal Hospital, Eilat, Israel; 5 Department of Orthopedic Surgery, E. Wolfson Medical Center, Holon, Israel

**Keywords:** Biodegradable spacer, Massive rotator cuff tears, Biceps tenotomy, Pain, Shoulder function, Constant score

## Abstract

**Background:**

Lesions of the long head of the biceps (LHB) tendon are frequently associated with massive rotator cuff tears (RCT) and may be responsible for shoulder pain and disability.

**Objective:**

This study aimed to evaluate functional outcomes of arthroscopic biodegradable spacer implantation with or without biceps tenotomy as treatment for persistent shoulder dysfunction and pain due to a massive irreparable RCT.

**Methods:**

A total of 48 patients were implanted with the subacromial spacer using arthroscopic approach with or without biceps tenotomy. All patients were assessed for up to 12 months post-implantation and 18 patients were assessed for at least 24 months (and a maximum of 40 months). Improvement in shoulder function was assessed using Constant score.

**Results:**

Subacromial spacer implantation was performed arthroscopically in 48 patients. The mean total Constant score increased from 36 at baseline to 67 points at 12 months post implantation. Patients who underwent LHB tenotomy in addition to the subacromial spacer presented similar improvement of their shoulder function and score compared to the group that was treated with the spacer alone.

**Conclusion:**

Current study demonstrates that spacer implantation in this patient population provides significant improvement in function and decreases the pain. Additional LHB tenotomy did not influence the postoperative results during the follow-up.

## 1 INTRODUCTION

Massive, degenerative rotator cuff (RC) tears can be disabling due to severe pain and/or weakness of the shoulder. Patients with massive RC tears often suffer from severe daily pain, poor sleep quality, and are disable to independently perform a common activities of daily living (ADL) [1, 2]. Many palliative interventions have been offered to patients suffering from massive/irreparable RC tear. Several surgical treatment modalities have been described for treating irreparable massive RCT, including simple debridement [3-5], tuberoplasty [6-8], tendon transfer [9, 10], tendon allograft [11] and prosthetic replacement [12, 13].

The long head of the biceps (LHB) tendon has been proposed as a source of pain in patients with RC tears [14, 15]. Based on many clinical studies, isolated arthroscopic biceps tenotomy or tenodesis is considered as an acceptable option for the treatment of RC tears in particular patients [14, 15]. Although it does not improve shoulder strength, tenotomy or tenodesis reported to reduce pain and improve the functional range of motion with a high degree of patient satisfaction [14, 15].

The latest treatment modality suggested for massive irreparable RCT is the InSpace™ system (Ortho-Space, Caesarea, Israel), implanted between the humeral head and the acromion [16-20]. The spacer is made of a biodegradable copolymer (Poly L-lactide-co-ε-caprolactone). It is inserted into the subacromial space and filled with a physiological solution (NaCl) to spread it in situ. This spacer deflates within the first four [4] months after introduction and fully degrades at ~12 months post implantation, which is the required period for post-operative shoulder rehabilitation. Clinical studies on patients with irreparable RC tears have suggested that the InSpace™ system can clinically reduce pain, enable physiotherapy and restore the range of motion and function in the long-term [17-19].

The purpose of the current study was to evaluate the clinical outcomes in a consecutive series of patients who had been treated with a biodegradable spacer with or without arthroscopic biceps tenotomy for persistent shoulder pain and dysfunction due to an irreparable massive RC tear.

## 2 PATIENTS AND METHODS

### 2.1 Study Design

This prospective multicenter study was conducted in accordance with the approved research protocol and good clinical practice guidelines. Five participating sites enrolled a total of 58 patients.

All subjects had persistent pain and functional disability for at least 4 months, imaging confirmation of a RC tear by either ultrasound, CT or MRI, and documented the failure of conservative therapy. Evidence of significant shoulder osteoarthritis, glenohumeral instability or active shoulder infection, previous shoulder surgery, uncontrolled diabetes mellitus, immunosuppression or coagulopathy were exclusion criteria.

For the purpose of diagnosis, RC tears were categorized as either Full Thickness (FT) or Partial Thickness (PT). The extent of the tear was classified intraoperative as small (<1 cm), medium (1-3 cm), large (3-5 cm), or massive (>5 cm) [20].

A total of 48 patients were implanted with the InSpace™ device in 4 different clinical sites using arthroscopic approach, the remaining 10 patients in an additional single site, were implanted using fluoroscopy guided technique under local anesthesia which was introduced as a feasibility sub-group for an implantation technique . Although this group was not significantly different in its results of the arthroscopy treated patients, it was excluded from the current data presentation and discussion due to the different technical modality. Of the 48 arthroscopic procedures, two patients underwent partial tear repair and the InSpace™ device was inserted over the repair. These two patients were also excluded from the presented efficacy analysis.

### 2.2 Surgical Technique

**Fig. (1):**
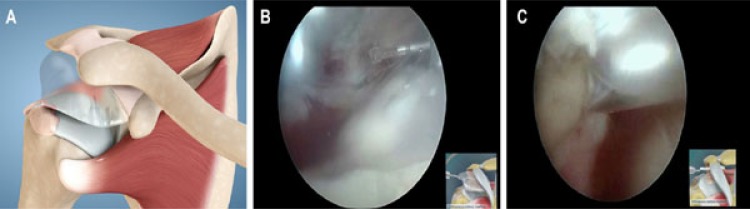
**A**- Schematic presentation of the spacer system. **B-C**- Final steps of deployment and sealed spacer in right shoulder with patient in beach-chair position, arthroscope in posterior portal, and spacer in lateral portal.

The implantation was performed utilizing arthroscopic procedure with the patient in a beach-chair position under general anesthesia as was previously described and published [17-19] (Fig. **1**). Briefly, posterior and anterolateral portals were used for complete arthroscopic evaluation before implantation of the subacromial spacer InSpace^TM^ Balloon System (Orthospace, Caesarea, Israel). Debridement of the subacromial space was done just to allow the spacer insertion. No acromioplasty or coracoacromial ligament resection was performed.

To select the appropriate spacer size, the subacromial space was measured using an arthroscopic probe .An appropriate subacromial spacer (made of polylactic acid and epsilon-caprolactone) was introduced through the lateral portal and was inflated with saline solution as recommended by the manufacturer. The shoulder is then taken through a full range of motion to confirm the stability and proper placement of the implant.

Of the 48 arthroscopic procedures, 97% (46 patients) were diagnosed with Massive irreparable RC tear and were treated with device implantation with or without LHB tenotomy while 3% (2 patients) were excluded from the presented efficacy analysis, as they underwent partial repair in addition to the InSpace™ implantation, four additional patients did not comply with study protocol requirements.

### 2.3 Clinical Assessment

Primary evaluation endpoints were assessed by complications reported during follow-up period along with the improvement of shoulder function over time using the Constant score (CS) [21]. Parameters were recorded preoperatively (baseline) and postoperatively at 2 and 6 weeks and afterwards at 3, 6 and 12 month. The post-operative follow-up was started at 2 weeks post implantation to indicate early recovery and improvement in shoulder pain and functionality. Primary outcome was targeted at 12 months post-implantation with prolonged follow up, as applicable.

All patients were contacted to conduct an additional follow-up at 24 to 40 months, however, only 43% of the patients (18/42) were willing to return for this late follow-up. The main reasons for early withdrawal from the study follow-up were logistic issues such as distance from clinic and change in general health condition associated with co- morbidities which are not related to their shoulder disease.

### 2.4 Statistical Analyses

Study data were analyzed with the SAS^®^ version 9.2 (SAS Institute, Cary NC, USA). The values are expressed as mean ± standard deviation (SD). Categorical variables are presented as counts and percentages. The mean changes from baseline in total CS and adjusted CS and its subscales were determined using a repeated measures analysis variance model. P values < 0.05 were considered statistically significant with no adjustment for multiple testing.

## 3 RESULTS

Out of the total 42 patients who presented with irreparable rotator cuff tears, 21 (50%) underwent InSpace^TM^ implantation without addressing the LHB and the other 21 (50%) underwent InSpace^TM^ implantation combined with LHB Tenotomy. In five [5] out of the 21 cases, LHB was not addressed due to the complete rupture of the tendon. No LHB tenodesis was performed. The decision to perform biceps tenotomy was depended on tendon integrity and made intraoperatively by the surgeon.

**Fig. (2):**
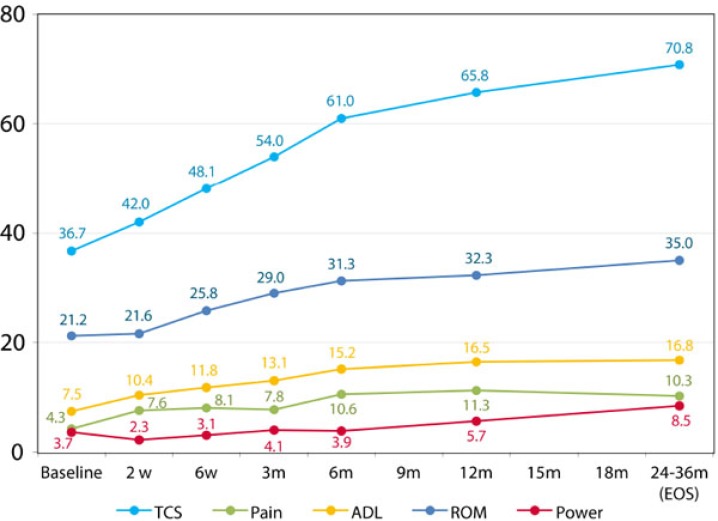
Graphical presentations of improvement of TCS over time at Protocol Population. Values are presented as means ±SD.

The Constant score improved throughout the study visits score of 36 at baseline to 67 at one year follow up (Fig. **2**). Apart from the power, all sub-scores of the Constant score including Pain, Activity of Daily Living (ADL) and Range of Motion (ROM) showed a statistically and clinically significant improvement throughout the study follow up from baseline to the 1 year follow up visit (Fig. **2**).

As presented in Fig. (**3**), the patients who underwent LHB Tenotomy in addition to the InSpace™ presented similar improvement of their shoulder function and scoring to the group that was implanted with the InSpace™ only.

**Fig. (4):**
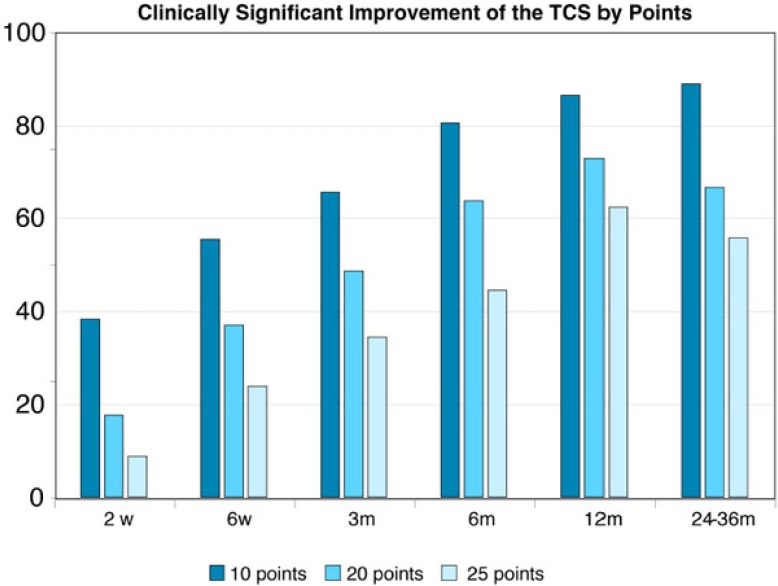
Graphical presentations of improvement in total CS during 2 years follow up. Values are presented as means ±SD.

Over 55% of the treated subjects showed clinical significant improvement of at least 10 points in TCS from 6 weeks’ post implantation onwards (Fig. **4**). At the 12 months follow up visit, over 85% of the evaluated subjects showed clinically significant improvement of at least 10 points in their TCS.

## 4 DISCUSSION

The principal results of this study demonstrated a significant improvement of the shoulder joint function in patients with massive and irreparable RC tears following implantation of the spacer. Unlike in previously published studies that reported a substantial improvement of the shoulder function following biceps tenotomy or tenodesis [14, 15], our results were not influenced by the aforementioned procedure. Additionally, these positive results were not influenced by factors such as severity of disease at baseline, age or gender.

No consensus regarding the ideal treatment option for patients suffering from massive/irreparable RC tears is yet available. On the basis of the force couple theory [3, 15], active motion can be possible despite massive RC tear if force coupling is maintained. However, some patients have complains about pain not responsive to conservative treatment even if active motion is conceivable. This study focused on patients with irreparable massive RC tears without significant degenerative changes of the glenohumeral joint or significant osteoarthritis.

LHB tendon pathologies are often associated with massive RC tears and have been identified as a source of persistent pain that can be resolved with spontaneous rupture of the tendon. Therefore, biceps tenotomy or tenodesis are considered as a treatment option for patients with massive and/or irreparable RC tears. To date, debates concerning this procedure have noted longevity of pain relief and a possible progression of arthritic changes in the glenohumeral joint.

Additionally, loss of shoulder flexion strength is often a concern for patients who are candidate for LHB sacrifice. However, arthroscopic biceps tenotomy in the treatment of RC tears in selected patients yields good objective improvement and a high degree of patient satisfaction. Walch *et al*. reported the long-term results of 307 biceps tenotomies, 110 of which were performed with a concomitant acromioplasty, as palliative treatment for RC tears [15]. At a mean of 57 months postoperatively, 87% of the patients were satisfied or very satisfied and the mean Constant score had improved to 67.6 points compared to 48 points pre-operatively. Our results are comparable with the results reported by Walch *et al*., demonstrating the Constant score improvement from 36 at baseline to 67 at one year following the spacer implantation.

Boileau **et al*.* evaluated the clinical and radiographic outcomes of isolated arthroscopic biceps tenotomy or tenodesis as treatment for irreparable RCT associated with a biceps lesion [22]. They concluded that pseudoparalysis of the shoulder and severe RC arthropathy are contraindications to this procedure.

However, Klinger *et al*. compared the results of arthroscopic debridement in massive, irreparable RC tears with and without tenotomy of the LHB in 41 cases [23]. In a line with our study results, they concluded that additional LHB tenotomy did not significantly influence the postoperative results at the latest follow-up. In addition, they did not mention any significant humeral head migration or development of rotator cuff arthropathy.

The current study presents similar results as a recently published articles which further established the continues experience with the device . Deranlot *et al*. reported that implantation of a subacromial spacer for MIRCT leads to significant improvement in shoulder function at the minimum 1 year postoperatively.

Their outcome measures included pre- and postoperative, range of motion, Constant score, acromiohumeral distance, and Hamada classification on anteroposterior and lateral radiographs [24].

Additional study by Ricci *et al*, concluded that the surgical procedure of the arthroscopic implantation of subacromial spacer for Massive Irreparable rotator cuff tears is an effective treatment modality that recovers the shoulder function with a reduction of the pain in worker patients and with recreational activities' demands [25].

This study has several limitations; small cohort of treated patients and the sole use of the change in total Constant score over time as an instrument for functional assessment. The follow up period was relatively short as the most significant change is expected to occur within the first 6 month following device implantation. This hypothesis was supported by Zuke *et al*. [26], who performed systematic review of 1370 patients who underwent rotator cuff repair. They presented that the major improvements in strength and range of motion as well as surgery related complications were seen up to 6 months post operation, but no clinically meaningful improvement was seen thereafter.

## CONCLUSION

In conclusion, we believe that after a conservative treatment had failed, in patients who had massive and irreparable RC tears, arthroscopic spacer implantation with or without biceps tenotomy appears to be a viable, palliative treatment option. Current study demonstrates that spacer implantation in this patient population provides significant improvement in function and decreases the pain. Additional LHB tenotomy did not influence the postoperative results during the follow-up. Further randomized controlled trials in larger cohorts of subjects assessing the clinical and functional outcomes after implantation of the InSpace™ device are warranted.

## Figures and Tables

**Fig. (3):**
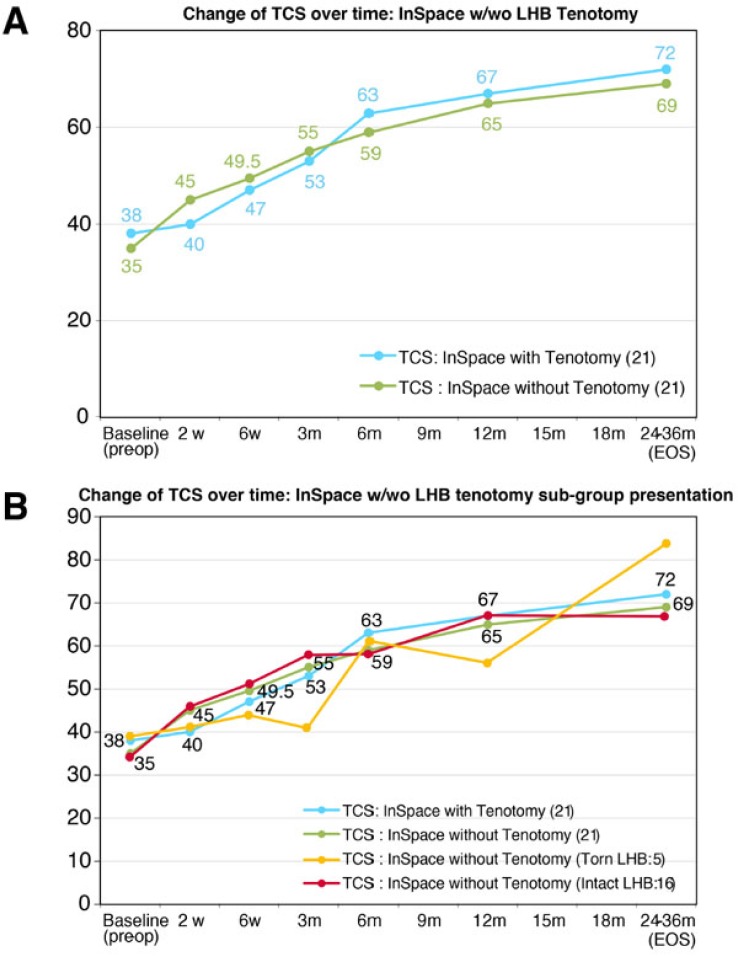
**A-** Change of TCS over time: InSpace™ w/wo LHB Tenotomy; **B**- Change of TCS over time: InSpace™ w/wo LHB tenotomy sub-group presentation. Values are presented as means ±SD.
